# Comparison of the Impact of Different Mass Drug Administration Strategies on Infection with *Schistosoma mansoni* in Mwanza Region, Tanzania—A Cluster-Randomized Controlled Trial

**DOI:** 10.4269/ajtmh.18-0671

**Published:** 2018-10-22

**Authors:** Annette Olsen, Safari Kinung’hi, Pascal Magnussen

**Affiliations:** 1 Department of Parasitology and Aquatic Pathobiology, Faculty of Health and Medical Sciences, University of Copenhagen, Copenhagen, Denmark;; 2 National Institute for Medical Research, Mwanza Research Centre, Mwanza, Tanzania;; 3 Centre for Medical Parasitology, Faculty of Health and Medical Sciences, University of Copenhagen, Copenhagen, Denmark

## Abstract

Annual school-based mass drug administration with praziquantel has been widely implemented to control schistosomiasis, but other treatment strategies could have a different impact. The aim of this study was to investigate the impact of six different treatment strategies on *Schistosoma mansoni* infection in a cluster-randomized controlled trial in schoolchildren, in a high transmission area of the Mwanza Region, Tanzania. A total of 150 villages were randomized into six arms with 25 villages in each arm. In each village, approximately 100 schoolchildren aged 9–12 years were randomly selected each year and investigated for *S. mansoni* prevalence and intensity based on three consecutive stool samples using the duplicate Kato–Katz technique. Four years of community-wide treatment (CWT) was the most intensive treatment strategy, whereas 2 years of school-based treatment (SBT) combined with 2 years without treatment (holiday) was the least intensive treatment. The remaining strategies constituted different combinations of CWT, SBT, and holiday years. Baseline results on *S. mansoni* infection were obtained from 14,620 schoolchildren from 148 villages, and mean prevalence and mean intensity among infected were 48.6–60.6% and 130.5–229.8 eggs per gram, respectively. Over the years, mean prevalence and mean intensities declined in all arms, but when comparing year 5 mean prevalence and mean intensity, there were no statistically significant differences between treatment arms. Thus, measured in a random selection of schoolchildren aged 9–12 years, four times CWT was not superior to four times SBT, while 2 years of treatment holiday combined with 2 years of SBT had the same impact as 4 years of SBT.

## Introduction

Schistosomiasis is caused by trematodes of the genus *Schistosoma* residing within the blood vessels of the host. The infection is especially widespread in countries of sub-Saharan Africa, and in Tanzania, both the urogenital and intestinal form of schistosomiasis are endemic.^1^ In the Mwanza Region bordering Lake Victoria, the intestinal form caused by *Schistosoma mansoni* is particularly abundant.^2,3^

Mass treatment with praziquantel has for decades been the main strategy for the control of schistosomiasis, and especially, school-based mass drug administration (MDA) has been widely implemented. However, after many years of school-based treatment (SBT) and with transmission still ongoing, the question arose whether other treatment strategies could differ in their impact. In other words, is community-wide (including schools) MDA more effective than school-based MDA alone? Or would 2 years of school-based MDA followed by, or alternating with, 2 years without treatment be just as effective as annual school-based MDA? To answer these questions, the Schistosomiasis Consortium for Operational Research and Evaluation (SCORE) Gaining Control projects were initiated in 2010. The aim was to compare the effect of different treatment strategies on *S. mansoni* and *Schistosoma haematobium* prevalence and intensity in 9- to 12-year-old schoolchildren over a 5-year period (four treatment rounds) in several different countries.^4^ The overall goal of the SCORE project is to provide evidence on how best to gain control of schistosomiasis infections in endemic areas.

This study reports the results of a multiyear, multi-arm cluster-randomized intervention trial in an area of the Mwanza Region, Tanzania, where baseline prevalence of *S. mansoni* in the individual villages was 25% or more. The development of mean prevalence and mean intensity from baseline to year 5 are reported for each treatment strategy, but the primary research question was as follows: Does the final year (year 5) mean prevalence and mean intensity of schistosomiasis among children aged 9–12 differ by study arm? Of the different potential comparisons, our main interest was to compare whether four times community-wide treatment (CWT) was more effective compared with four times SBT when measured in a random selection of a group of 9- to 12-year-old schoolchildren. Our secondary interest was to investigate what impact 2 years without treatment, either alternatively or consecutively, combined with 2 years of SBT would have on *S. mansoni* prevalence and intensity compared with 4 years of SBT.

## Materials and Methods

### Ethics statement on subject recruitment.

The study was reviewed and approved by the Medical Research Coordination Committee of the National Institute for Medical Research (NIMR), Tanzania (ethics clearance certificate no. NIMR/HQ/R.8a/Vol.IX/1022), and the University of Georgia Institutional Review Boards, Athens, GA (2011-10353-1). Before examination and sample collection, the reasons for the survey and the procedures of sample collection were explained to the children and the adult population in the communities including local leaders, school administration, teachers, and health and education personnel. The trial was registered with ClinicalTrials.gov (NCT02162875) and International Standard Randomized Controlled Trial 95819193. Only children who assented to participate and had written informed consent from parents or legally authorized representatives were eligible for inclusion and were requested to give stool samples.

### Study area and population.

**Figure 1.**
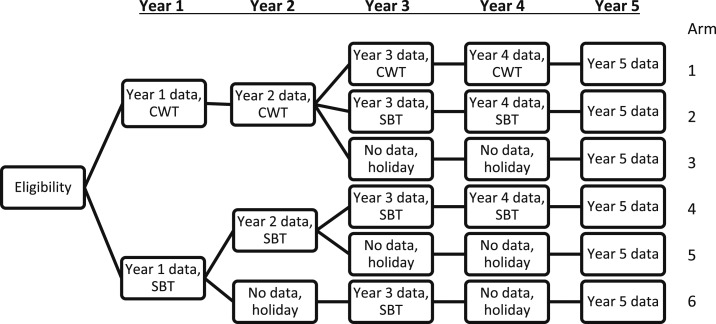
Study design. Villages were randomized into six intervention arms. Holiday indicates years in which a village did not receive mass drug administration with praziquantel. For ethical reasons, children’s infection status was not investigated during drug holiday years. CWT = community-wide treatment; SBT = school-based treatment.

The study was carried out from August 2011 to September 2016 in villages that had at least one school within a 10-km distance from Lake Victoria in Geita, Sengerema, Misungwi, Nyamagana, Ilemena, and Magu districts, Mwanza Region, Tanzania. Transmission of *S*. *mansoni* in this area is considered perennial and a prior site selection survey had identified villages with a prevalence of *S*. *mansoni* of 25% or more in 13- to 14-year-old schoolchildren. At study start, the region had 1,139 primary schools with 902,367 schoolchildren enrolled, which is more than 95% of all school age children in the region.^5^ A total of 150 villages were randomized into six arms with 25 villages in each arm using a simple randomization procedure without stratification. In each of these villages, one school was selected (in case there was more than one school), and 100 schoolchildren between the age of 9 and 12 years were randomly selected each year with new selections each year. The primary outcomes of interest were *S. mansoni* prevalence and intensity among these children (Figure 1). The study protocol of this study with detailed descriptions of sample size calculations is described in a study by Ezeamama et al.^6^

### Stool sample collection, examination, and treatment.

Participants were given stool containers and asked to bring fresh stool specimens to the school on three consecutive days. Stool samples were immediately processed in the school using duplicate Kato–Katz thick smears with a 41.7-mg template from each specimen.^7^ All slides were transported to the NIMR in the Mwanza Region and examined for *S. mansoni* eggs. Slides were marked with village and child identification number but did not reveal treatment arm. Infection with soil-transmitted helminths were not investigated as *Ascaris lumbricoides* and *Trichuris trichiura* infections have been shown to occur with a very low prevalence^8,9^ and because eggs of the more prevalent hookworm infection would not be visible because of the long time span between preparation and reading.

School-based treatment was performed in the school and included all school-aged children (enrolled and non-enrolled children) for treatment. Village health workers helped in identifying the non-enrolled children. Community-wide treatment targeted the whole community and treatment was performed at a central place (school, health facility, or market place). Treatment was performed by the study team annually (expect in villages on drug holidays) and information on day of treatment was given to schools and communities at least 7 days in advance of treatment.

### Statistical analysis.

Data were analyzed using IBM statistics SPSS version 24 (IBM, Armonk, NY). A person was considered positive for infection if at least one egg was found in any of the slides. Mean egg counts of the six slides were calculated and multiplied by 24 to express the intensity as eggs per gram of stool (epg). Six slides were obtained in 92.4% of all investigated cases, but in those cases where some slides were missing, the calculation was performed on available slides. Group intensity was computed as the arithmetic mean of epg from the total number of investigated persons (village-level intensity) and of the infected persons only (individual-level intensity).

As village is the clustering factor, mean prevalence was calculated as the means of the prevalence of individual villages. Likewise, village-level arithmetic mean intensity and individual-level arithmetic mean intensity were calculated as the means of the mean of individual villages. The Mann–Whitney *U*-test was used to assess differences in mean prevalence and mean intensities between baseline and year 5 because data were not normally distributed and because different study populations were investigated in the 2 years.

Individual-level intensity was divided in categories according to the World Health Organization guidelines^10^ as light: 1–99 epg; moderate: 100–399 epg; and heavy: ≥ 400 epg. The sum of these three infection levels will inevitably present the overall prevalence for each arm not accounting for village as the clustering factor. The overall prevalence is not appropriate for the used study design and will only be shown in the figure presenting intensity levels.

The treatment coverage of schoolchildren in the village was calculated by dividing the number of treated children of the age of 9–12 years with the number of all the children in the village in the same age group (including both enrolled and non-enrolled children). The treatment coverage of the community, for those villages receiving community-based treatment, was calculated by dividing the number of treated persons with the total population eligible for treatment (registered by annual updated census data). Pregnant women were excluded but lactating women were eligible and treated.

Comparisons of end prevalence and end intensities (village-level intensity) in year 5 between arms were analyzed using generalized estimating equations. Unadjusted estimates—with just village and arm fitted in the model—and adjusted estimates—where gender and age were also included in the model, along with weighting for number of children providing data are reported. All statistical tests used were two sided, and *P* < 0.05 was considered significant.

Protocol deviations were discovered in six villages. Two villages swapped arms throughout the whole study (from arms 2 to 5, and vice versa) and four villages in three arms (arms 3, 5, and 6) received treatment during one of the holiday years. A sensitivity analysis was carried out to account for these deviations by repeating the analysis with these six villages removed, but the removal of the six villages did not have an impact on the findings, and hence, the results reported are for all villages.

## Results

In 2010, a site selection study identified 150 villages with *S. mansoni* prevalence of 25% or more. During the screening, 50 children aged 13–14 years were investigated in each of 308 villages. The study flow by arm is shown in the Supplemental Figure 1. A total of 149 villages were included because one village in arm 1 has to be excluded because of low cooperation.

Table 1 shows the baseline characteristics of 9- to 12-year-old schoolchildren by study arm. In addition to the permanently excluded village in arm 1, one village in arm 3 did not provide data for year 1 and was excluded from year 1 only. Mean prevalence was between 48.6% and 60.6% and individual-level arithmetic mean intensity was between 130.5 and 229.8 epg in the six arms.

**Table 1:** Baseline characteristics of participants by study arm

	Arm 1 (cccc)*	Arm 2 (ccss)*	Arm 3 (cchh)*	Arm 4 (ssss)*	Arm 5 (sshh)*	Arm 6 (shsh)*
Number of villages (*n* = 148)	24	25	24	25	25	25
Number of participants (*n* = 14,620)	2,455	2,404	2,313	2,496	2,585	2,367
Mean age in years (range)	10.6 (9–12)	10.7 (9–12)	10.5 (9–12)	10.5 (9–12)	10.7 (9–12)	10.7 (9–12)
Percentage of girls	51.6	53.8	54.9	53.4	53.2	51.2
Number infected	1,384	1,353	1,143	1,309	1,547	1,381
Mean prevalence in % (range)†	56.9 (9.3–100)	55.5 (6.0–98.0)	48.6 (7.2–99.0)	52.5 (4.6–98.7)	60.6 (14.4–100)	57.6 (16.3–100)
Village-level arithmetic mean infection intensity in epg (range)‡	151.0 (2.8–525.6)	98.4 (1.2–379.5)	127.0 (4.7–775.8)	112.5 (1.0–619.5)	180.3 (7.0–1,138.2)	113.6 (3.3–545.3)
Individual-level arithmetic mean infection intensity in epg (range)§	201.4 (30.0–525.6)	130.5 (11.8–427.9)	184.6 (27.0–799.8)	162.0 (17.8–627.6)	229.8 (30.9–1,138.2)	148.0 (18.0–549.8)

epg = eggs per gram.

* c = community-wide treatment; h = drug holiday year; s = school-based treatment.

† Calculated as means of the prevalence of individual villages.

‡ Calculated as means of the means of individual villages, all investigated children included.

§ Calculated as means of the means of individual villages, only infected children included.

Mean prevalence (means of the prevalence of individual villages) were similar for females and males within each arm for all 5 years (Supplemental Table 1). Hence, Table 2 shows the results at baseline and in year 5 for all six arms for females and males combined. Only four times CWT showed a significant decrease in mean prevalence from baseline to year 5. However, the individual-level mean intensity was significantly reduced from baseline to year 5 in all arms except for arm 2 (CWT, CWT, SBT, and SBT).

**Table 2:** Descriptive results for 9- to 12-year-old schoolchildren at baseline and year 5

	Arm 1 (cccc)*	Arm 2 (ccss)*	Arm 3 (cchh)*	Arm 4 (ssss)*	Arm 5 (sshh)*	Arm 6 (shsh)*
No. tested at baseline	2,455	2,404	2,313	2,496	2,585	2,367
No. infected at baseline	1,384	1,353	1,143	1,309	1,547	1,381
Means of the prevalence of individual villages at baseline in %†	56.9	55.5	48.6	52.5	60.6	57.6
No. tested at year 5	2,358	2,481	2,476	2,420	2,484	2,468
No. infected at year 5	942	1,081	1,193	1,033	1,234	1,207
Mean of the prevalence of individual villages at year 5 in %†	40.0	43.6	47.9	42.0	49.3	48.6
Absolute difference between mean prevalence at year 5 and baseline in %	−16.9	−11.9	−0.7	−10.5	−11.3	−9.0
Relative difference between mean prevalence at year 5 and baseline in % change	29.7	21.4	1.4	20.0	18.6	15.6
*P*-value of difference between mean prevalence at year 5 and baseline‡	0.035	0.15	0.87	0.14	0.20	0.27
Village-level arithmetic mean infection intensity at baseline, epg§	151.0	98.4	127.0	112.5	180.3	113.6
Village-level arithmetic mean infection intensity at year 5, epg§	40.8	44.3	58.6	43.7	77.6	53.5
Egg reduction rate (100% (1-year 5 arithmetic mean/year 1 arithmetic mean)	73.0	55.0	53.9	61.2	57.0	52.9
Individual-level arithmetic mean infection intensity at baseline, epg‖	201.4	130.5	184.6	162.0	229.8	148.0
Individual-level arithmetic mean infection intensity at year 5, epg‖	81.4	77.4	98.8	80.8	122.6	75.8
*P*-value of difference between individual-level mean intensity between year 5 and baseline‡	0.003	0.14	0.041	0.035	0.032	0.024

epg = eggs per gram.

* c = community-wide treatment; h = years of holiday; s: school-based treatment.

† Calculated as means of the prevalence of individual villages.

‡ Mann–Whitney *U*-test.

§ Calculated as means of the means of individual villages, all investigated children included.

‖ Calculated as means of the means of individual villages, only infected children included.

**Figure 2.**
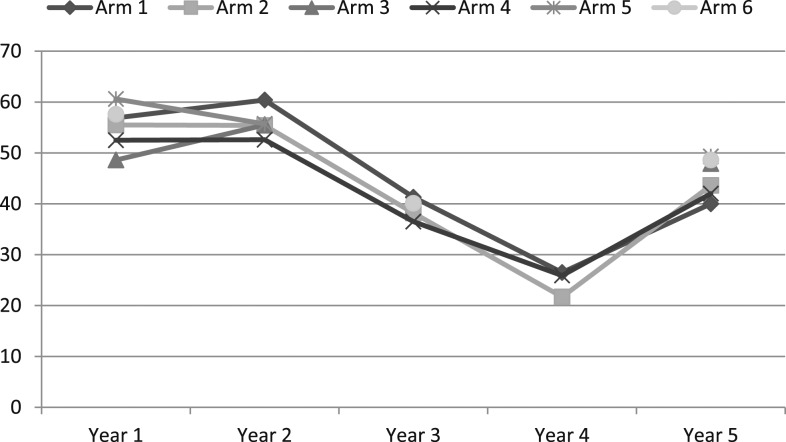
Changes in mean prevalence calculated as means of the prevalence of individual villages in % by year by arm for all children between the age of 9 and 12 years.

Figure 2 shows the changes in mean prevalence (means of the prevalence of individual villages) for females and males combined from years 1 to 5 for the different arms. The decrease from years 1 to 5 was only significant in arm 1 (see Table 2). All arms showed the same pattern with a decrease from years 2 to 4 followed by an increase in year 5. The increase from years 4 to 5 in those three arms which were investigated in the 2 years was significant in arm 1 (*P* = 0.047) and arm 2 (*P* = 0.005), and approached significance in arm 4 (*P* = 0.071).

**Figure 3.**
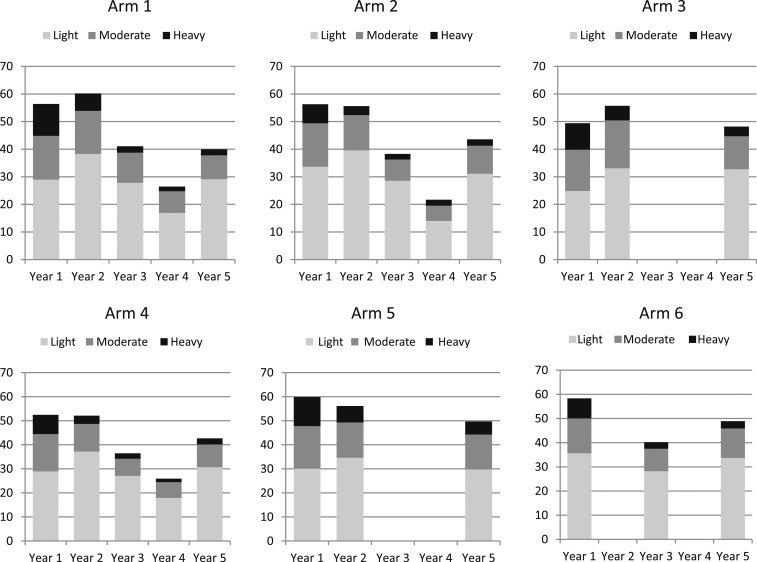
Intensity categories by year for each arm for all children aged 9–12 years (both genders combined) showing the percentage of light (1–99 eggs per gram [epg]), moderate (100–99 epg), and heavy (≥ 400 epg) infection intensities. The height of the columns represents the overall prevalence, which is different from the mean prevalence of individual villages presented in Table 2 and Figure 2.

In Figure 3, the intensity categories by year and by arm are shown for all children aged 9–12 years (both genders combined) as the percentage of light, moderate, and heavy infection intensities are similar for females, males, and the total population. As stated previously, the height of the columns (the sum of the three infection levels) represents the overall prevalence, which is different from the mean prevalence (means of the prevalence of individual villages) presented in Table 2. In year 5, the percentages of moderate to heavy infections were reduced to 10.9%, 12.5%, 15.5%, 12.1%, 19.9%, and 15.2% in arms 1–6, respectively.

The treatment coverage of school age children was calculated for all arms for years 1 to 4 and was between 72.2 and 83.2% (Table 3). Community-wide treatment coverage was between 76.7 and 81.5%. By protocol, treatment coverage of 75% or higher was considered acceptable.

**Table 3:** Coverage: proportion of children aged 9–12 years treated by arm by year among enrolled and non-enrolled children, and proportion of total population in community treated among the population eligible for treatment

	Arm 1 (cccc)*		Arm 2 (ccss)*		Arm 3 (cchh)*		Arm 4 (ssss)*	Arm 5 (sshh)*	Arm 6 (shsh)*
	Children	Community	Children	Community	Children	Community	Children	Children	Children
Year 1	75.7	76.7	78.5	79.3	74.0	80.1	82.6	81.6	83.2
Year 2	75.8	79.4	80.2	81.5	77.0	81.5	82.4	80.3	NA^2^
Year 3	74.6	77.1	72.2	NA^1^	NA^2^	NA^2^	76.9	NA^2^	78.1
Year 4	74.7	76.7	76.1	NA^1^	NA^2^	NA^2^	80.5	NA^2^	NA^2^

NA^1^ = not applicable because only school-based treatment was carried out in years 3 and 4; NA^2^ = not applicable because testing was not performed in years when villages were not receiving treatment.

* c = community-wide treatment; h = drug holiday year; s = school-based treatment.

Comparisons of end prevalence and end intensities (village-level intensity) in year 5 among arms are shown in Table 4. The number of comparisons is restricted with a focus on the comparison of the current standard treatment in arm 4 (four times SBT) with the most obvious alternative treatment strategies. The primary question was whether four times CWT in arm 1 was superior to arm 4. A secondary question was whether SBT for 2 years with 2 years of treatment holiday in arms 5 and 6 (either as holiday every other year or 2 years of holiday at the end) was just as effective as SBT for 4 years (arm 4). Apart from these three comparisons, 2 years of CWT followed by 2 years of SBT (arm 2) and 2 years of CWT followed by 2 years of holiday (arm 3) were compared with 4 years of CWT (arm 1). None of these five comparisons were significantly different.

**Table 4:** Comparisons of year 5 prevalence and year 5 village-level intensity (all investigated children) of *Schistosoma mansoni* infection in 9- to 12-year-old children between selected arms

Comparison*	Prevalence				Intensity			
	Unadjusted prevalence ratio (95% CI)	*P* value	Adjusted prevalence ratio (95% CI)	*P* value	Unadjusted intensity ratio (95% CI)	*P* value	Adjusted intensity ratio (95% CI)	*P* value
Arm 4 vs. arm 1 (ssss vs. cccc)	1.1 (0.6–2.0)	0.80	1.0 (0.5–2.0)	0.88	1.1 (0.6–2.0)	0.83	0.9 (0.5–1.9)	0.91
Arm 2 vs. arm 1 (ccss vs. cccc)	1.2 (0.6–2.2)	0.65	1.2 (0.6–2.2)	0.61	1.1 (0.6–2.1)	0.80	1.1 (0.6–2.1)	0.78
Arm 3 vs. arm 1 (cchh vs. cccc)	1.4 (0.7–2.5)	0.29	1.4 (0.8–2.5)	0.27	1.4 (0.7–2.7)	0.28	1.4 (0.7–2.6)	0.35
Arm 5 vs. arm 4 (sshh vs. ssss)	1.3 (0.7–2.6)	0.39	1.4 (0.7–2.8)	0.37	1.8 (0.9–3.3)	0.069	1.9 (0.9–3.6)	0.055
Arm 6 vs. arm 4 (shsh vs. ssss)	1.3 (0.7–2.6)	0.44	1.3 (0.7–2.7)	0.39	1.2 (0.6–2.5)	0.58	1.3 (0.6–2.7)	0.49

c = community-wide treatment; Cl = confidence limits; h = years without treatment (holiday); s = school-based treatment.

## Discussion

In this cluster-randomized trial, we compared the impact of different treatment strategies on *S. mansoni* infection among 9- to 12-year-old children attending schools in an area where baseline infection prevalence was 25% or more. The overall finding was that all treatment strategies reduced mean prevalence and mean intensities, although not statistically significant throughout. However, when comparing mean prevalence and mean intensity at year 5, there was no statistically significant difference between the six different treatment strategies in this high transmission area.

Thus, when measured in 9- to 12-year-old schoolchildren, four times CWT did not result in significantly lower prevalence or intensities of infection compared with four times SBT, which is considered the standard strategy. On the other hand, having 2 years of treatment holiday in between (or after) 2 years of SBT had the same impact as four times SBT, which is consistent with the finding in a low-to-moderate prevalence area of Kenya.^11^ These data suggest that biennial SBT may have similar benefits as annual SBT in schistosomiasis control programs in both low-to-moderate and high transmission areas. This has important implications as it will be attractive to conserve donated praziquantel tablets. Changing from annual to biennial SBT in an area means that the amount of praziquantel available could cover treatment of twice as many children in the same time period. However, as we anticipate that the optimal treatment strategy for a given area is dependent on a multitude of local factors, program managers must ensure that using a strategy including years without treatment will have support from the affected communities.

When looking at the decrease in the prevalence of infection from baseline to year 5 in individual arms, the four times CWT was the only strategy which showed a statistically significant decrease (29.7%), while the decrease was insignificant in all other arms, or even negligible in arm 3, which received 2 years of CWT followed by 2 years of treatment holiday. This emphasizes that school-based MDA alone will not be sufficient to eliminate schistosomiasis and that health authorities have to focus on the aquatic snail hosts and other essential components for elimination such as safe water supplies, adequate sanitation and proper health education.^12^

The decrease from baseline to year 5 in infection intensity of the infected children was significant for all treatment strategies with the exception of the arm receiving twice CWT and twice SBT (arm 2). There seems not to be a rational explanation for this because arms 3–6 received less intense treatment compared with arm 2 and these other strategies resulted in significant decreases. However, in all arms, it was possible to reduce the percentage of heavy and moderate infections to below 20% (Figure 3).

The significant increase in mean prevalence from years 4 to 5 observed in our study was unexpected and likely reflects increased transmission following the year 4 MDA and the El Nino rains during that time. In year 4, MDA was implemented between May and June 2015, whereas the year 5 parasitological survey was performed during March-September 2016. Precipitation was normal and almost zero in the first part of this period from May to September 2015, but from September 1, 2015, to September 1, 2016, the cumulative precipitation for the Mwanza Region was 2,000 mm compared with the normal 1,000 mm.^13^ This means that water could be collected in ponds and puddles near the lakeshore creating new possible transmission habitats increasing the general force of transmission in the area. In contrast to this, precipitation was normal in 2012, 2013, and 2014, where data for years 1–3 were collected. This year 5 increase has obviously impacted the magnitude of the prevalence reductions between baseline and year 5 for all treatment arms.

There were some limitations in our study. It was not possible in all schools to reach the target of 100 children in the right age group, but the mean at baseline was between 94.7 and 103.4 in the six arms, resulting in a total of 14,620 children; only 2.5% lower than the targeted 15,000. However, the target was reached in years 2–4 but ended at the same level in year 5 as during baseline.

The treatment coverage of 75% was achieved in most arms and years with the exception of schoolchildren in arm 1 (years 3 and 4), arm 2 (year 3), and arm 3 (year 1). However, the lowest coverage was 72.2% in arm 2, which is only 3.7% lower than the target. Whether the low coverage has contributed to the lack of a significant reduction in individual-level intensity from years 1 to 5 in arm 2 is not known but is of course a possibility.

Despite the target of the site selection study to identify villages/schools with a *S. mansoni* prevalence of 25% or more, 30 of the 149 schools (20%) had children at the age of 9–12 years with lower than 25% prevalence at baseline. The likely explanation for this is that the site selection study screened children aged 13–14 years and that children of that age have higher prevalence and intensity than their 9- to 12-year-old peers. As other schools had very high prevalence (up to 100%), it did not impact the mean prevalence but resulted in a very wide variation around the mean prevalence, which likely reduced the power to detect significant differences between arms. Another factor that contributed to this wide variation around mean prevalence and intensity during follow-up years was unexpected village/school variability in terms of MDA responsiveness within arms. Some villages/schools decreased substantially in prevalence and intensity, whereas other villages/schools failed to decrease to expected levels of these parameters. The definition and discussion of these persistent hotspots are reported previously.^14^

In conclusion, this large-scale study provides data that show that intensity can be decreased by any of the strategies, even in the face of sporadic years with high transmission rates, but it could not determine one MDA treatment strategy as favorable over another. When compared at year 5, four times CWT was not superior to four times SBT when measured in a random selection of schoolchildren aged 9–12 years. On the other hand, 2 years of treatment holiday combined with 2 years of SBT had the same impact as 4 years of SBT. As stated previously, this means that biennial SBT could be implemented if the local community accepts this. However, there is a need to focus on other strategies if the goal is to achieve reductions in prevalence and intensity needed to break transmission (i.e., elimination). This could be to treat more frequently than annually and to implement additional interventions such as snail control, safe water supply, adequate sanitation, and health education.

## Supplementary Material

Supplemental figure
