# Supplementary material for: Comparison of the Impact of Different Mass Drug Administration Strategies on Infection with Schistosoma mansoni in Mwanza Region, Tanzania—A Cluster-Randomized Controlled Trial
Source: Am J Trop Med Hyg. 2018 Oct 22;99(6):1573–9. doi: 10.4269/ajtmh.18-0671 (PMC6283472; doi:10.4269/ajtmh.18-0671)
Supplement: Supplementary file 1 [file tpmd180671.SD1.pdf]

Figure S1. Study flow chart

Screened for eligibility (n= 308); excluded (n=158) for not meeting inclusion criteria; included 150 villages

Enrollment (2010) and Randomization (2011)

Arm	No. of villages (Baseline)	Number of participants per village	
		Mean	SD
1 [1]	24	102.3	5.3
2	25	96.2	19.8
3 [2]	24	96.4	16.2
4	25	99.8	10.9
5	25	103.4	6.6
6	25	94.7	15.7

2012

Arm	No. of villages (year 2)	Number of participants per village	
		Mean	SD
1 [1,3]	23	104.8	5.5
2	25	105.0	5.5
3	25	103.8	5.5
4	25	103.2	5.3
5	25	104.4	6.5
6	-	-	-

2013

Arm	No. of villages (year 3)	Number of participants per village	
		Mean	SD
1 [1]	24	99.7	2.9
2	25	98.4	6.8
3	-	-	-
4	25	99.8	2.9
5	-	-	-
6	25	101.4	3.9

2014

Arm	No. of villages (year 4)	Number of participants per village	
		Mean	SD
1 [1]	24	100.0	0
2	25	100.0	0
3	-	-	-
4	25	100.0	0
5	-	-	-
6	-	-	-

2015

Arm	No. of villages (year 5)	Number of participants per village	
		Mean	SD
1 [1]	24	98.3	6.3
2	25	99.2	5.8
3	25	99.0	2.8
4	25	96.8	8.2
5	25	99.4	5.0
6	25	98.7	2.7

Final data collection (2016)

[1] Nyakasungwa, Tz120 was excluded due to low compliance (all years)

[2] Ihale, Tz040, no children was investigated in Y1

[3] Bukumbi, Tz009 was missing in Y2

Table S1. Mean prevalence calculated as means of the prevalence of individual villages in % by year by arm for females and males separately and for the two sexes combined. Difference between genders are calculated using the Mann-Whitney U Test

	Arm 1	Arm 2	Arm 3	Arm 4	Arm 5	Arm 6
**Year 1**						
**Females**	53.3	52.4	47.3	50.1	58.2	57.4
**Males**	60.6	58.6	50.0	55.1	63.4	57.8
**Total**	56.9	55.5	48.6	52.5	60.6	57.6
***P*-value for difference between genders**	0.32	0.37	0.87	0.36	0.57	0.94
**Year 2**						
**Females**	59.5	54.8	55.2	52.8	56.2	-
**Males**	61.1	56.1	56.1	52.4	55.8	-
**Total**	60.4	55.4	55.5	52.6	55.7	-
***P*-value for difference between genders**	0.87	0.89	0.99	0.84	0.92	
**Year 3**						
**Females**	39.6	35.3	-	35.3	-	39.5
**Males**	43.2	42.2	-	37.8	-	40.8
**Total**	41.3	38.2	-	36.5	-	40.1
***P*-value for difference between genders**	0.59	0.46		0.57		0.66
**Year 4**						
**Females**	24.7	20.8	-	24.5	-	-
**Males**	28.9	22.9	-	27.8	-	-
**Total**	26.5	21.7	-	25.9	-	-
***P*-value for difference between genders**	0.38	0.99		0.52		
**Year 5**						
**Females**	40.3	42.4	46.3	39.6	48.6	47.1
**Males**	39.7	44.9	49.6	45.0	50.4	50.1
**Total**	40.0	43.6	47.9	42.0	49.3	48.6
***P*-value for difference between genders**	0.98	0.73	0.64	0.66	0.89	0.72
